# Binaural interaction component in adults with normal hearing

**DOI:** 10.1016/j.bjorl.2026.101844

**Published:** 2026-06-16

**Authors:** Mariana de Carvalho Leal, Adilane de Sousa Costa Reis, Lilian Ferreira Muniz

**Affiliations:** Universidade Federal de Pernambuco, Graduate Program in Human Communication Health, Recife, PE, Brazil

**Keywords:** Binaural, Electrophysiology, Hearing, Auditory evoked potentials

## Abstract

**Objective:**

This study aimed to identify the binaural interaction component in adults with normal hearing through the use of the Auditory Brainstem Response (ABR).

**Methods:**

A cross-sectional, observational, quantitative study was conducted at a philanthropic hospital specialized in Otorhinolaryngology and Speech-Language Pathology, located in Real Hospital Português de Beneficência em Pernambuco. The study was approved by the Research Ethics Committee (approval nº 6.203.668). The sample consisted of 20 adults, aged 19–52 years, of both sexes, with normal hearing. Participants underwent auditory brainstem response testing to calculate the binaural interaction component. Stimuli were presented in the following sequence: right monaural, left monaural, and then binaural stimulation. Descriptive analysis was performed using the mean of the recorded potentials. The binaural response was calculated by subtracting the sum of the monaural responses from the binaural response for each potential. Inferential analysis was conducted using the Mann-Whitney *U* test.

**Results:**

The binaural interaction component was observed in all waves of the auditory brainstem response, with more consistent findings in wave V. No significant statistical differences were found when correlating the results with the studied variables. However, a trend was observed toward reduced wave amplitudes with increasing age.

**Conclusion:**

Adults with normal hearing present the binaural interaction component, as demonstrated through auditory brainstem response testing.

**Level of Evidence:**

Level 4.

## Highlights

• Binaural Interaction Component is an objective method for analyzing binaurality.
• The BIC reflects the ability to recognize and integrate different auditory stimuli.
• Auditory Evoked Potentials are neuroelectric activities of the auditory pathway.

## Introduction

Hearing is a highly valuable sense that enables us to perceive sound waves and, consequently, to interact with all the sounds around us. This process occurs binaurally, through both ears; thus, binaurality refers to the ability of the Central Auditory Nervous System (CANS) to receive auditory information from both ears, integrate, analyze, and combine them, thereby facilitating the comprehension of different sounds, particularly in noisy environments.

The focus of this study is the Binaural Interaction Component (BIC), an objective measure of binaurality assessed through electrophysiological auditory tests, and its objective is to identify the binaural interaction component in adults with normal hearing using the auditory brainstem evoked potential. The BIC represents the gain or improvement in responses calculated by subtracting the sum of the monaural electrophysiological responses from the binaural response.^1^

Binaural interaction ability can also be investigated through Auditory Evoked Potentials (AEPs), from which the binaural interaction component can be derived. Although there is scientific evidence supporting this approach, it is mainly established for brainstem-level potentials using specific types of stimuli.^2^ It is important to emphasize that binaural abilities are acquired over time, as the nervous system matures and with increasing auditory experience.^3^^,^^4^

The BIC reflects the capacity to recognize and integrate different auditory stimuli presented asynchronously but complementarily or sequentially to both ears.^5^ In practice, the BIC can be calculated as the Binaural Response (BIN) minus the sum of the Right monaural (*R*) and Left monaural (*L*) responses, expressed algebraically as: BIC = BIN - (R + L).^6^

Expanding knowledge about the assessment of the BIC through electrophysiological tests in adults with normal hearing is essential, as it contributes to the establishment of normative standards. Among the examinations used to investigate the integrity and maturation of auditory pathways, Auditory Evoked Potentials (AEPs) are the most frequently applied.

The establishment of normative data for the Binaural Interaction Component (BIC) has important clinical relevance, as it provides reference parameters for the objective assessment of central auditory pathways. These data can be used to identify alterations in binaural integration in individuals with normal hearing and to support the early diagnosis of auditory disorders, as well as the planning and monitoring of speech-language pathology interventions.

Furthermore, the availability of normative standards contributes to the standardization of clinical procedures and future research, allowing reliable comparisons across different populations and studies. Thus, the results of this study provide valuable information that may serve as a basis for future research and as a reference for clinical practice.

## Methods

A cross-sectional, observational, quantitative study was conducted in the Audiology Department of a private philanthropic hospital recognized as a reference center in Audiology and Otorhinolaryngology, located in Real Hospital Português de Beneficência in Pernambuco, Brazil. The study followed ethical guidelines and was approved by the institution’s Research Ethics Committee (REC) for Human Subjects under approval number 6.203.668.

### Sample and participant selection

The sample consisted of 20 adults, including 12 females (60%) and 8 males (40%), aged between 19- and 52-years. Participants were recruited by convenience from patients and companions attending the service, in accordance with the previously established inclusion and exclusion criteria.

The sample size was determined by convenience, taking into account the feasibility of the study and the availability of participants who met the strict inclusion criteria. This careful selection aimed to ensure sample homogeneity and the reliability of the electrophysiological recordings, although it resulted in a relatively small sample size.

### Inclusion criteria

Normal otoscopy; pure-tone audiometry thresholds ≤ 25 dB HL at frequencies between 250 Hz and 8000 Hz; type A tympanometric curve; presence of ipsilateral and contralateral acoustic reflexes; presence of Auditory Brainstem Response (ABR); and voluntary consent to participate in the study.

### Exclusion criteria

Abnormal results in any of the above-mentioned tests (outside normative standards); self-reported or clinically confirmed neurological disorders at the time of the interview; difficulty in understanding the tasks, compromising proper execution of the tests; and history of exposure to noise or intense sound (above 80 dB HL) either regularly or within 72 h prior to assessment, to avoid potential alterations in auditory thresholds during audiometry.

The volunteers recruited were patients or companions of patients attending the clinic. They were invited by the researcher to participate in the study, informed about its objectives, and asked to sign the Informed Consent Form. All participants were fully briefed on the testing procedures to ensure correct understanding of the instructions and were informed about the average duration of the data collection process. Those who agreed to participate in the study signed the Informed Consent Form (ICF) and completed an interview (questionnaire) providing personal and audiological information.

### Data collection

Participants underwent otoscopy to rule out external ear or ear canal abnormalities that could affect the results of subsequent examinations. Conventional audiological assessment was then performed, including pure-tone and speech audiometry, as well as immittance measurements. Finally, Auditory Brainstem Responses (ABR) were recorded, and the Binaural Interaction Component (BIC) was analyzed.

Potentials were initially recorded in the monaural condition, starting with the right ear, with a minimum of 1000 stimuli. Recording was then performed for the left ear, followed by binaural stimulation using the same number of stimuli. Table 1 describes the protocol used for ABR recording.

**Table 1:** Protocol used for Auditory Brainstem Response (ABR) recording.

ABR Parameters	Values
Stimulus	Click
Polarity	Rarefied
Intensity (dB nHL)	19.1
Averaging	1000
High-pass Filter	30 Hz
Low-pass Filter	3000 Hz
Gain	100,000
Pre-stimulus Analysis Time	−1 ms
Analysis Time	12 ms

Each wave was recorded twice; duplication of each recording ensured reproducibility and reliability of the waves. Wave markings, which characterize the responses, were identified by two experienced examiners, and in case of disagreement, a third examiner was consulted.

### Statistical analysis

Data were compiled and tabulated using Microsoft Excel 365, and statistical analyses were performed using SPSS 26.0 (Statistical Package for the Social Sciences) for Windows and Excel 365. Descriptive and quantitative statistical analyses were conducted. Variables are presented as mean ± Standard Deviation (SD).

Descriptive statistics were obtained for the analysis of wave latencies and amplitudes. Comparisons between ears and other variables were conducted using the Mann-Whitney *U* test, a non-parametric test. All tests were applied with a 95% Confidence Interval.

The Mann-Whitney *U* test is used to assess whether two independent samples originate from populations with equal means. This test is an alternative to the independent-samples *t*-test when the sample size is small and/or the assumptions required for the *t*-test are violated. The Mann-Whitney test was chosen due to the small sample size, which is considered to be fewer than 30 participants (n < 30).

Waves I, III, and V were analyzed with respect to absolute latencies (ms) and amplitudes (μV). Latency measurements were obtained by identifying the wave peaks in the electrophysiological trace, considering the most positive point of the wave deflection as the latency peak. Amplitudes were calculated as the difference between the wave peak and the immediately preceding trough. All recordings were evaluated by two experienced examiners, and in cases of disagreement, a third evaluator was consulted.

## Results

The sample consisted of 20 adults, including 12 women (60%) and 8 men (40%), with a mean age of 32.05-years (range: 19–52 years). The mean age was 32.05-years, with a standard deviation of 9.21-years and a median of 31-years.

For statistical convenience, the sample was divided into two subgroups: adults under 40-years of age and adults aged 40-years and above, as shown in Table 2.

**Table 2:** Characteristics of the study population (n = 20).

Variables	n	%
Age		
<40-years	13	65.0
≥40-years	7	35.0
Male	8	40.0
Female	12	60.0

The Table 3 presents the means, medians, minimum and maximum values of the absolute latencies and their respective Standard Deviations (SD) for waves I, III, and V of the 40-ears evaluated, regardless of sex or side.

**Table 3:** Means and standard deviations of absolute latencies (ms) and BIC amplitudes (μV) (n = 40-ears)^a^.

	Wave I	Wave III	Wave V
Mean	0.28	0.40	0.52
SD	±0.35	±0.84	±1.18
Median	0.23	0.22	0.31
Min–Max	1.46–0.27	3.68–0.37	5.39–0.30

a Mann-Whitney test.

In Table 4, the means of the latencies and amplitudes of waves I, III, and V are presented according to the mode of presentation (monaural and binaural). Longer latencies were observed in the right ear, and higher amplitudes for waves I and V in the left ear during monaural listening. When comparing binaural to monaural listening, longer latencies were noted for waves III and V, as well as a higher amplitude for wave V.

**Table 4:** Means of latencies and amplitudes of waves according to mode of presentation (monaural and binaural)^a^.

Monaural Listening						
	Wave I		Wave III		Wave V	
Ear Right	Mean	SD	Mean	SD	Mean	SD
Latency	1.82	±0.24	3.86	±0.22	5.76	±0.28
Amplitude	0.27	±0.35	0.44	±0.82	0.65	±1.19

	Wave I		Wave III		Wave V	
Ear Left	Mean	SD	Mean	SD	Mean	SD
Latency	1.66	±0.31	3.69	±0.54	5.74	±0.27
Amplitude	0.32	±0.18	0.23	±0.23	0.45	±0.16

Binaural Listening						
	Wave I		Wave III		Wave V	
	Mean	SD	Mean	SD	Mean	SD
Latency	1.75	±0.16	3.79	±0.29	5.75	±0.23
Amplitude	0.30	±0.18	0.27	±0.17	0.57	±0.18

I, First wave; III, Third wave; V, Fifth wave.

a Mann-Whitney test.

In this study, as shown in Table 5, no significant differences were observed regarding the variable age; however, there was a trend toward decreased amplitude with increasing age.

**Table 5:** Comparison of mean sums of wave amplitudes and Binaural Interaction Component (BIC) obtained with monaural and binaural presentation according to age^a^.

<40 years						
	Wave I		Wave III		Wave V	
	Meam	SD	Meam	SD	Meam	SD
Binaural	0.32	±0.20	0.30	±0.19	0.58	±0.19
Monoaural	0.34	±0.16	0.22	±0.21	0.45	±0.17
BIC	−0.36	±0.39	−0.46	±0.31	−0.69	±1.44
p-value	0.165		0.721		0.427	

≥40 years						
	Wave I		Wave III		Wave V	
	Meam	SD	Meam	SD	Meam	SD
Binaural	0.26	±0.17	0.20	±0.09	0.56	±0.15
Monaural	0.28	±0.22	0.24	±0.27	0.44	±0.14
BIC	−0.15	±0.24	−0.30	±0.34	−0.21	±0.27
p-value	0.165		0.721		0.427	

I, First wave; III, Third wave; V, Fifth wave. BIC, Binaural Interaction component.

a Mann-Whitney test.

In Table 6, when comparing males and females according to the mode of listening presentation, higher responses were observed for waves I and V in binaural listening for males. The other means did not show relevant differences. The table also presents standard deviations for these means and the corresponding p-values.

**Table 6:** Comparison of mean sums of wave amplitudes and Binaural Interaction Component (BIC) obtained with monaural and binaural presentation according to sex^a^.

Male						
	Wave I		Wave III		Wave V	
	Meam	SD	Meam	SD	Meam	SD
Binaural	0.31	±0.20	0.23	±0.12	0.61	±0.17
Monoaural	0.25	±0.12	0.24	±0.16	0.48	±0.14
BIC	−0.20	±0.14	−0.26	±0.26	−0.34	±0.13
p-value	0.189		0.699		0.395	

Female						
	Wave I		Wave III		Wave V	
	Meam	SD	Meam	SD	Meam	SD
Binaural	0.29	±0.18	0.29	±0.19	0.55	±0.18
Monaural	0.31	±0.18	0.39	±0.15	0.60	±0.18
BIC	−0.34	±0.44	−0.50	±1.08	−0.64	±1.53
p-value	0.189		0.699		0.395	

I, First wave, III, Third wave; V, Fifth wave; BIC, Binaural Interaction Component.

a Mann-Whitney test.

Comparisons between ears, modes of presentation (monaural vs. binaural), sex, age, and educational level were performed using the Mann-Whitney test. None of the observed differences between groups reached statistical significance (p > 0.05). All non-significant results are presented as such, without any interpretative suggestion of an effect or trend.

For example, when comparing the mean amplitudes of waves I, III, and V between the right and left ears, no significant differences were found (p = 0.189, 0.699, and 0.395, respectively). Similarly, analyses by age group (< 40-years vs. ≥ 40-years) and by educational level (undergraduate vs. postgraduate/master’s) revealed no statistically significant differences (p > 0.05 for all parameters).

## Discussion

This study demonstrates that the Binaural Interaction Component (BIC) was identified in all participants with normal hearing, across all ABR waves, with wave V being the most consistent. No statistically significant differences were found regarding age, sex, or educational level (p > 0.05 for all parameters). All non-significant results were clearly presented, without any interpretative suggestion of trends or effects.^7^

Studies on brainstem auditory evoked potentials with binaural stimulation have shown that the amplitude of the sum of monaural recordings is greater than that obtained from the binaural response.^5^ In this study, all analyses of amplitudes related to the sum of monaural recordings were higher than those of binaural recordings, corroborating the findings of Skoe.^5^

Regarding amplitude and sex, the male group exhibited higher wave V amplitudes compared to females, consistent with previous literature reporting increased wave V amplitude in men.^8^^,^^9^ These differences may represent a potential source of bias, especially in studies with small sample sizes, and should be considered in future comparisons.

According to Eddins,^10^ further electrophysiological studies are needed to investigate the relationship between binaural processing and age, as aging is known to degrade the perception of binaural signals, but little is known about how neural representation of these signals is affected. Studies indicate that age influences ABR component latencies, with latencies increasing with age even in the absence of neurological disorders. Additionally, another study using ABR reported altered recordings in elderly individuals.^11^

The results of this study showed that responses to binaural stimulation presented smaller wave amplitudes than the sum of monaural responses. The electrophysiological traces for waves I, III, and V were clearly visible, and the mean absolute latencies were prolonged for wave V in both monaural stimulation for each ear and binaural stimulation.

The limited number of participants was due to the adoption of strict inclusion criteria, necessary to ensure normal hearing and the reliability of the recorded responses. Auditory evoked potentials can be influenced by physiological noise, muscle relaxation, environmental noise, and electrical interference. Part of these limitations was related to the small sample size, as delays in data collection impacted the acquisition of electrophysiological recordings.

Although the results obtained were consistent and aligned with the literature, future studies are recommended to include larger samples and explore the Binaural Interaction Component (BIC) in different clinical contexts, in order to contribute to the establishment of more robust normative standards and a better understanding of individual variations.

## Conclusion

This study demonstrates that adults with normal hearing exhibit the Binaural Interaction Component (BIC) when assessed using Auditory Brainstem Responses (ABR). The BIC was identified in all participants and across all ABR waves. Overall, the most consistent responses were observed in wave V.

None of the variables studied showed statistically significant differences. The study underscores the exploratory nature of the findings, acknowledging limitations related to the small sample size. The results provide important preliminary information that can serve as a foundation for future research and as a reference for clinical practice.

## ORCID ID

Mariana de Carvalho Leal: 0000-0002-1472-9586

## Funding

I declare that the submitted manuscript did not receive any financial support from any source, and neither I, the co-authors, nor the study participants have any financial or other interests related to the subject addressed in the manuscript.

## Data availability

The authors declare that all data are available in repository.

## Declaration of competing interest

I, Adilane de Sousa Costa Reis, corresponding author of the manuscript “Binaural Interaction Component in Adults with Normal Hearing”, declare that none of the authors of this study have any of the interests described below, or any other interests that could be considered a Conflict of Interest.
